# Hemophilia A gene therapy via intraosseous delivery of factor VIII-lentiviral vectors

**DOI:** 10.1186/s12959-016-0105-1

**Published:** 2016-10-04

**Authors:** Carol H. Miao

**Affiliations:** 1 Seattle Children’s Research Institute, Seattle, WA USA; 2 Department of Pediatrics, University of Washington, Seattle, WA USA

**Keywords:** Hemophilia A, Factor VIII, Gene therapy, Intraosseous delivery, Lentiviral vectors, Megakaryocyte-specific gene expression, Anti-FVIII inhibitory antibodies, Stem cell gene therapy

## Abstract

Current treatment of hemophilia A (HemA) patients with repeated infusions of factor VIII (FVIII; abbreviated as *F8* in constructs) is costly, inconvenient, and incompletely effective. In addition, approximately 25 % of treated patients develop anti-factor VIII immune responses. Gene therapy that can achieve long-term phenotypic correction without the complication of anti-factor VIII antibody formation is highly desired. Lentiviral vector (LV)-mediated gene transfer into hematopoietic stem cells (HSCs) results in stable integration of FVIII gene into the host genome, leading to persistent therapeutic effect. However, ex vivo HSC gene therapy requires pre-conditioning which is highly undesirable for hemophilia patients. The recently developed novel methodology of direct intraosseous (IO) delivery of LVs can efficiently transduce bone marrow cells, generating high levels of transgene expression in HSCs. IO delivery of E-F8-LV utilizing a ubiquitous EF1α promoter generated initially therapeutic levels of FVIII, however, robust anti-FVIII antibody responses ensued neutralized functional FVIII activity in the circulation. In contrast, a single IO delivery of G-FVIII-LV utilizing a megakaryocytic-specific GP1bα promoter achieved platelet-specific FVIII expression, leading to persistent, partial correction of HemA in treated animals. Most interestingly, comparable therapeutic benefit with G-F8-LV was obtained in HemA mice with pre-existing anti-FVIII inhibitors. Platelets is an ideal IO delivery vehicle since FVIII stored in α-granules of platelets is protected from high-titer anti-FVIII antibodies; and that even relatively small numbers of activated platelets that locally excrete FVIII may be sufficient to promote efficient clot formation during bleeding. Additionally, combination of pharmacological agents improved transduction of LVs and persistence of transduced cells and transgene expression. Overall, a single IO infusion of G-F8-LV can generate long-term stable expression of hFVIII in platelets and correct hemophilia phenotype for long term. This approach has high potential to permanently treat FVIII deficiency with and without pre-existing anti-FVIII antibodies.

## Background

Deficiency of blood clotting factor VIII (FVIII) results in hemophilia A (HemA), a serious bleeding disorder. Current treatment of HemA patients with repeated infusions of FVIII is costly, inconvenient, and incompletely effective [1]. In addition, approximately 25 % of treated patients develop anti-FVIII immune responses. Gene therapy that can achieve long-term phenotypic correction without the complication of anti-FVIII antibody formation represents a highly desirable approach to treat HemA patients.

Previous phase I gene therapy clinical trials [2–4], however, produced only transient, low-level FVIII expression due to inefficient gene delivery and induction of immune responses to FVIII and/or gene therapy vectors. The hematopoietic stem cells (HSCs) in bone marrow (BM) can serve as a significant target for stable integration of therapeutic genes into the genome. Therapeutic levels of FVIII have been obtained by ex vivo gene therapy using HSCs transduced by retroviral vectors carrying porcine FVIII combined with immune suppression and busulfan [5, 6]. However, it is highly undesirable to perform pre-conditioning for hemophilia patients.

It is demonstrated recently that in vivo gene transfer can be successfully carried out by direct intraosseous (IO) injection using several different vectors including adeno-, retro-, and lenti-viral vectors (LVs) [7–10]. HSCs can be efficiently transduced by these vectors and the transgene expression was detected in both progenitors and differentiated cell lineages [7, 8, 11]. This in vivo protocol corrected BM defects for long-term in diseased animals with Fanconi anemia [11]. Many drawbacks of ex vivo gene therapy, including maintenance of stem cell properties, low levels of engraftment, and side effects of cytokine stimulation can be evaded [9, 10, 12]. Most importantly, no pre-conditioning of the subject is required for this approach, thus providing a novel strategy for treating HemA.

In this concise review, we will discuss the recently developed novel approach of IO delivery of LVs to correct hemA [10]. The benefit and limitations of using LVs driven by ubiquitous and megakaryocyte-specific promoters will be compared. The potential of the development of this novel in vivo technology into clinically feasible gene transfer protocol to treat hemA patients, especially the clinically challenging patients with pre-existing inhibitory antibodies will be discussed.

## Review

### Gene therapy vs. protein replacement therapy and other therapies

Current treatment of hemophilia involves repeated infusions of FVIII protein either as regular prophylaxis or treatment during bleeding episodes. For severe patients, the standard treatment consists of intravenous infusion of factor VIII concentrates three times per week or every other day [6, 7]. In addition, 25 % of the patients develop inhibitory antibodies to FVIII following repeated infusions of FVIII. In recent years, efforts have been made to improve the efficacy of protein replacement therapy. One of the major successes is to prolong the half-life of FVIII in circulation [13]. This is recently achieved by either attaching polyethylene glycol (PEG) to FVIII (PEGylated FVIII) [14, 15] or fusing a monomeric Fc fragment of immunoglobulin G [16] or albumin [17] to FVIII. Less frequent infusions of FVIII can be administered to patients with these long lasting FVIII proteins. Another successful approach is the development of a humanized bispecific antibody (emicizumab; ACE910) that binds to activated factor IX and factor X and mimics the cofactor function of FVIII [18, 19]. In the first clinical trial, the patients were given subcutaneous emicizumab weekly for 12 weeks. The bleeding rate decreased significantly in hemophilia patients with and without inhibitory antibodies. Nonetheless, these therapies still need frequent infusions of costly reagent and the long-term side effects such as formation of antibodies against the products themselves still need to be evaluated over time. Compared to drug or protein therapy, gene therapy can achieve a prolonged therapeutic effect with only one or a few treatment for the lifetime.

### Comparison between different viral vector-mediated gene therapies for hemophilia

Recently, clinical trials of gene delivery of factor IX mediated by adenovirus-associated viral (AAV) vectors into the liver generated encouraging results in hemophilia B patients [20–22]. Furthermore, AAV-mediated, liver-directed gene transfer of FVIII produced therapeutic levels of gene expression in mice, dogs or macaques [23–25]. Most recently, Biomarin reported encouraging results of phase I/II clinical trial in a small number of patients using AAV5-mediated gene transfer. Five out of six high dose patients showed FVIII levels ranging between 4 and 60 % [26]. However it was noted there was elevation of ALT liver enzyme levels and a prophylactic corticosteroid therapy was given. The duration of clinical benefit in the treated patients remains to be determined. Furthermore, AAVs persist as episomal, concatemerized vectors following in vivo gene transfer, whereas transgene expression cassettes integrate into the host genome following LV transduction, leading to long-term therapeutic effect. If successful, a single treatment of LV-mediated gene therapy will be sufficient for life-long effect. Additionally, one key advantage of LV over traditional integrating gamma retroviral vectors is that it can transduce both non-dividing and dividing cells, leading to significantly increased efficiency targeting primitive stem cells. Furthermore, addition of SIN LTR elements in self-inactivating (SIN)-LVs provided improved safety by reducing transactivation capacity [27], thus permitting inclusion of enhancer-less internal promoters in the transgene expression cassettes [28]. In two recent clinical trials of ex vivo gene therapy for Wiskott-Aldrich Syndrome (WAS) [29] and Metachromatic leukodystrophy (MLD) [30], dramatic clinical improvement was obtained without adverse effects or aberrant clonal expansion. These results are very encouraging, suggesting that LV may be a safer gene therapy vector compared with traditional retroviral vector.

### In vivo HSC gene therapy mediates sustained transduction of HSCs in HemA mice

**Fig. 1:**
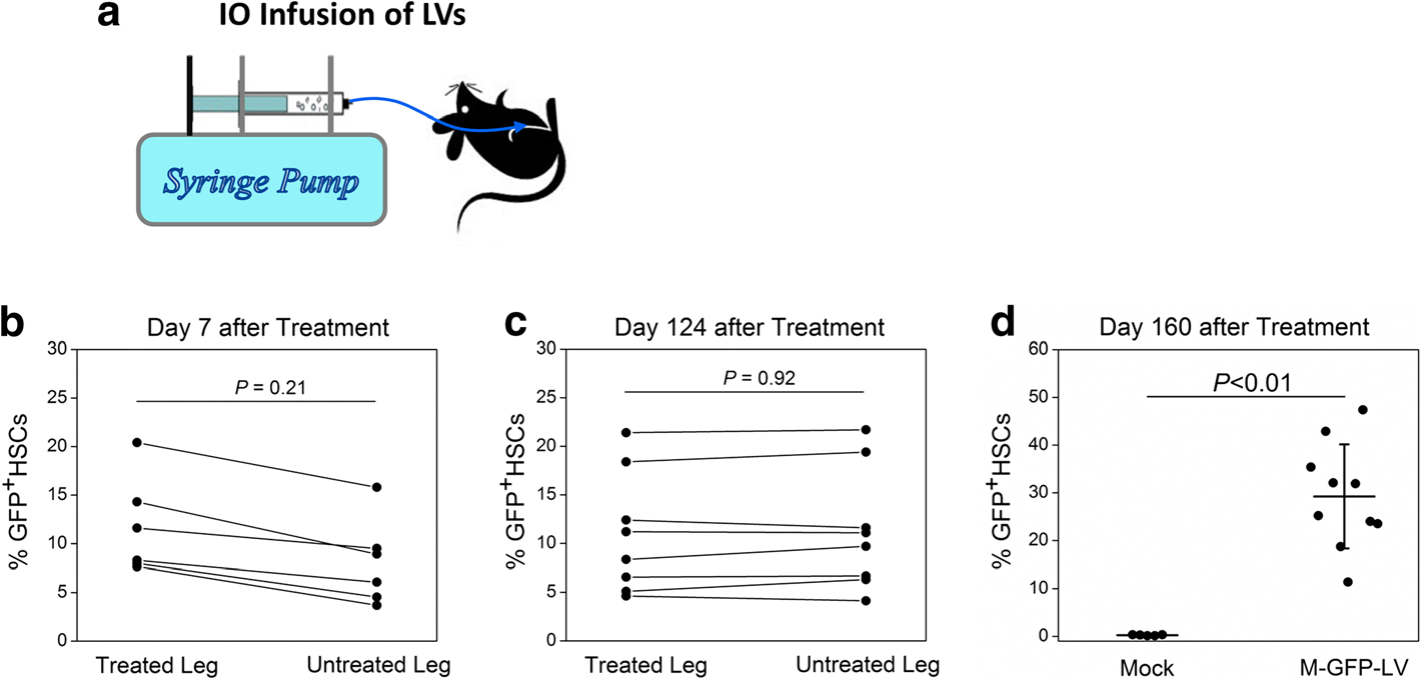
GFP expression in BM cells following IO infusion of M-GFP-LV. **a** Schematic of IO infusion of vectors into the mice with an infusion speed of 10 μl/min, which was precisely controlled by a programmable microfluidics syringe pump. **b** C57BL/6 mice were intraosseously delivered with M-GFP-LV (1.1 × 10^8^ ifu/animal, *n* = 6) on day 0. BM cells were isolated from treated or untreated legs and GFP expression in Lin^−^Sca1^+^c-Kit^+^ HSCs were examined on day 7 by flow cytometry. **c** C57BL/6 mice were given IO infusion of M-GFP-LV (1.1 × 10^8^ ifu; *n* = 8) on day 0. GFP expression in Lin^−^Sca1^+^c-Kit^+^ HSCs of treated and untreated legs was evaluated on day 124. **d** C57BL/6 mice were given IO infusion of M-GFP-LV (8.8 × 10^8^ ifu/animal; *n* = 10) or PBS (20 μl/animal, mock; *n* = 5) on day 0. Long-term GFP expression in Lin^−^Sca1^+^c-Kit^+^ HSCs was detected on day 160. This figure is reproduced from Ref [10]

Although ex vivo HSC transduction/transplantation protocols can successfully deliver FVIII into HemA mice [6, 31], the procedure requires pre-conditioning using potentially toxic, myelosuppressive agents. On the contrary, IO delivery to transduce HSCs in vivo can bypass this step, which is more desirable for treating hemophilia patients. Furthermore, no significant thrombocytopenia potentially induced by preconditioning regimens is expected with the IO delivery protocol. Additionally, compared with intravenous infusion of LVs into the circulation, IO delivery directly introduced LVs into the BM microenvironment, thus significantly enhancing the transduction efficiency of HSCs. In our recent report, a syringe pump was used to slowly infuse VSV-G pseudotyped SIN-LV vectors into the BM so that more vectors can be in better contact with the resident cells to achieve high levels of transduction [10] (Fig. 1a). When the BM cells were examined 7 days post infusion of M-GFP-LV driven by a ubiquitous MND promoter, a significant GFP signal was observed in HSCs of the treated mice (Fig. 1b). Furthermore, GFP expression persisted in 10–50 % HSCs up to 160 days (Fig. 1c&d), indicating that IO delivery of LVs has achieved efficient transduction of primitive progenitor cells in BM. Moreover, lower numbers of transduced cells were initially found in the untreated leg compared to the treated leg (Fig. 1b), whereas at later times, similar numbers of transduced cells were obtained at both sites (Fig. 1c), indicating that the transduced HSCs were transferred from treated to untreated BM sites over time. These results clearly demonstrated that IO LV delivery mediates sustained transduction of hematopoietic stem/progenitor cells [10, 32].

### Comparison between IO deliveries of F8-LVs driven by two different promoters

As mentioned in the introduction, one of the major complications of hemophilia treatment is the formation of anti-FVIII inhibitory antibodies. For achieving successful gene therapy, it is essential to prevent or evade anti-FVIII immune responses. For this purpose, platelet has been considered as a potential gene transfer vehicle to ectopically express FVIII because: (1) FVIII can be released to promote clot formation when the circulating platelets are recruited to and activated at the injury sites, (2) when needed, ectopic FVIII can be provided by circulating platelets that are produced daily from megakaryocytes in BM, (3) in particular, platelet FVIII stored in α-granules is not neutralized by anti-FVIII antibodies in the circulation [33, 34]. Previous reports showed that platelet-restricted expression of FVIII using megakaryocyte-specific promoters (glycoprotein (Gp) αIIb [31, 33, 34], Gp1bα [35] and platelet factor 4 [36]) can partially correct hemophilia phenotype in transgenic mice or in lethally irradiated HemA mice treated with ex vivo gene therapy. Ectopic expression of FVIII in platelets locally delivers protein and concomitantly evades anti-FVIII immune responses in treating HemA.

**Fig. 2:**
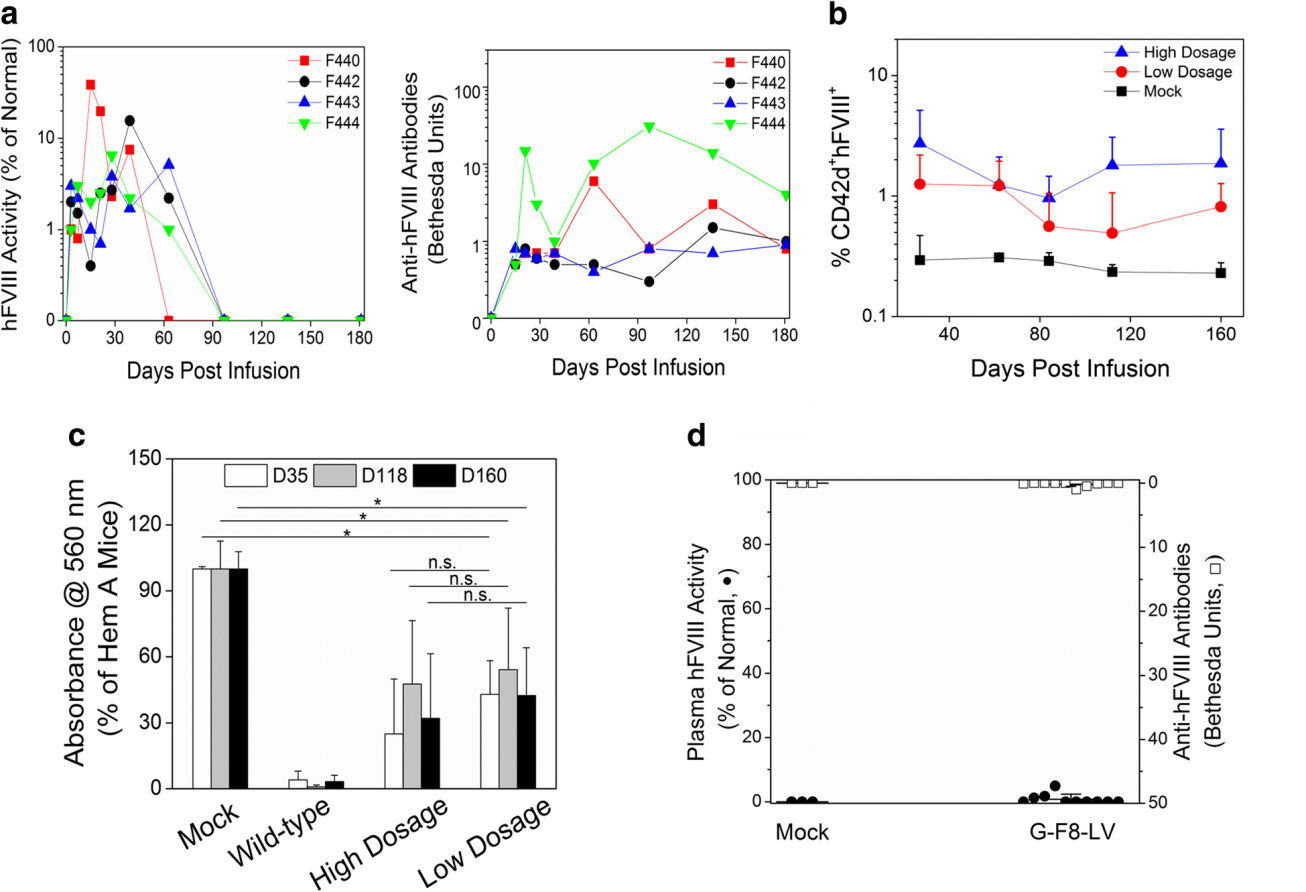
Comparison of hFVIII levels in plasma and/or platelets after a single IO infusion of E-F8-LVs and G-F8-LVs. **a** HemA mice were intraosseously infused with E-F8-LV (5 × 10^7^ ifu/animal, *n* = 4) or PBS (20 μl/animal, mock, *n* = 3) on day 0. Plasma samples were collected and hFVIII activity and anti-FVIII antibodies were measured by aPTT and Bethesda assay, respectively. No FVIII activity or anti-FVIII antibody was detected in the PBS treated control mice (data not shown). **b**-**d** HemA mice were given IO infusion of G-F8-LV (2.2 × 10^7^ ifu/animal or 2.2 × 10^6^ ifu/animal) or PBS (20 μl/animal, mock) on day 0. **b** Platelets were isolated from peripheral blood of high (*n* = 8) or low (*n* = 5) titer G-F8-LV treated or mock (*n* = 3) mice. hFVIII expression levels in CD42d^+^ platelets were evaluated by flow cytometry on day 27, 62, 84, 112 and 160. **c** HemA phenotype correction of G-F8-LV treated mice was monitored by tail clip assay on day 35, 118, and 160 (*n* = 4–7/group). The average blood loss of untreated HemA mice was set as 100 %. Wild-type C57BL/6 mice were used as positive controls. * *P* < 0.05. **d** Plasma samples were collected from high titer G-F8-LV treated (*n* = 10) or mock (*n* = 3) mice, and hFVIII activity and anti-FVIII antibodies were measured by aPTT and Bethesda assay, respectively. This figure is reproduced from Ref [10]

In our recent study [10], it was found that IO delivery of E-F8-LV using a ubiquitous human elongation factor-1α (EF1α) promoter into BMs of hemA mice efficiently transduced HSCs and produced initial high-levels of FVIII. However, a robust immune response to FVIII was induced, eliminating functional FVIII in the circulation (Fig. 2a). In contrast, a single IO delivery of G-F8-LV using a human megakaryocytic-specific GP1bα promoter produced persistent platelet-specific expression of FVIII (Fig. 2b), leading to persistent, partial correction of HemA phenotype (Fig. 2c). Interestingly, we did not detect hFVIII activity or anti-hFVIII antibody in plasma up to 160 days post treatment (Fig. 2d), implying that little or no hFVIII expression was secreted into the circulation. These results are consistent with previous work using the Gp1bα promoter to direct FVIII expression in platelets [35, 37]. It was shown that FVIII co-localized with VWF and stored in α-granules of platelets via a regulated secretary pathway [34].

### A single IO infusion of G-F8-LV produced persistent expression of FVIII in platelets

**Fig. 3:**
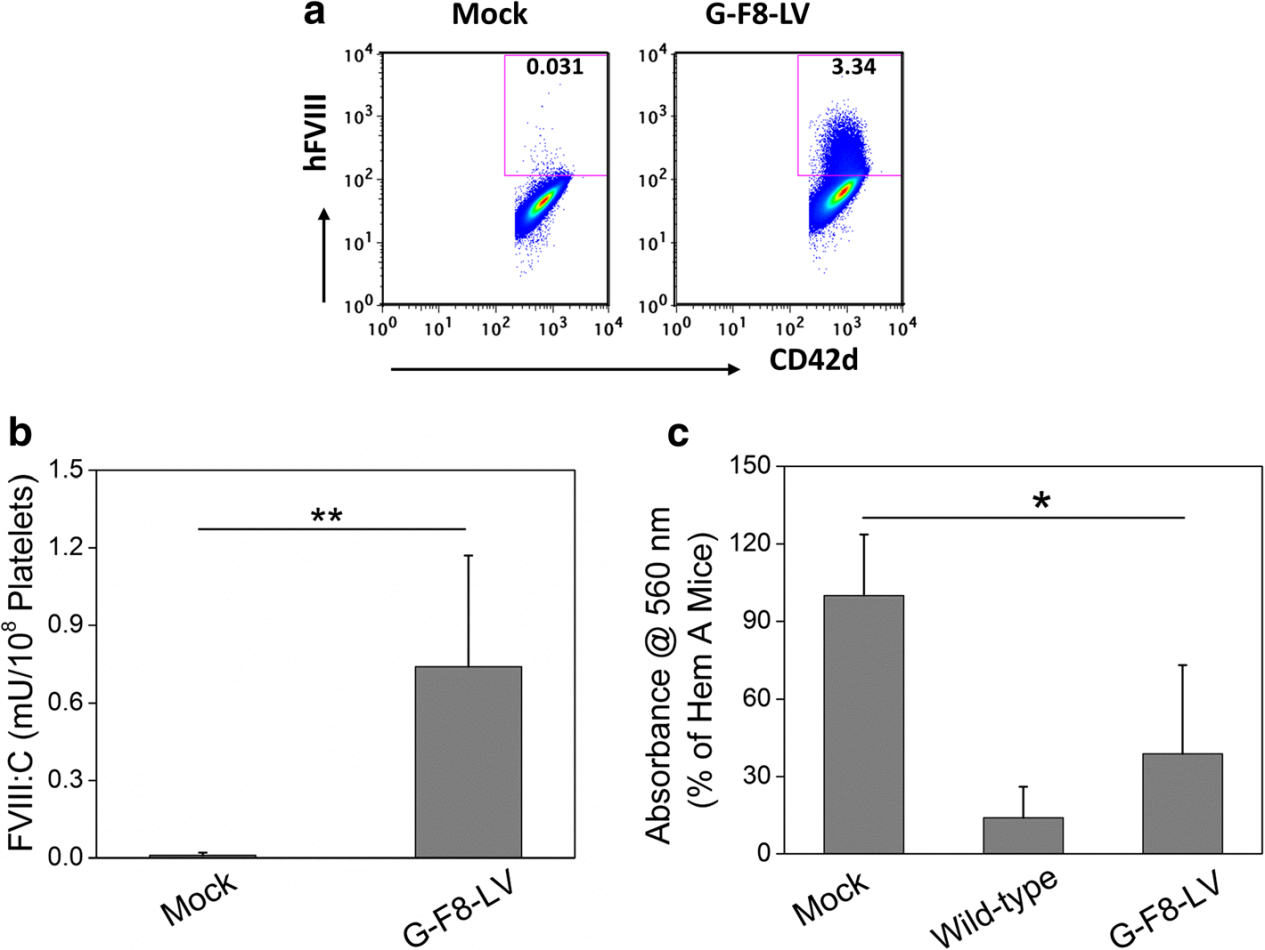
hFVIII expression in platelets of G-F8-LV treated inhibitor HemA mice corrected their hemophilia A phenotype. Inhibitor HemA mice were established by repeated intraperitoneal injection (3×/week for 2 weeks) of 3U rhFVIII into 10- to 12-week-old HemA mice. These inhibitor HemA mice were then intraosseously infused with G-F8-LV (2.2 × 10^7^ ifu/animal) or PBS (20 μl/animal, mock) on day 0. **a** Platelets were isolated from peripheral blood and marked with CD42d^+^, and their GFP expression levels at 5 months post infusion. **b** Platelets from LV-treated (*n* = 5) and mock (*n* = 3) mice and lysed. The resulting lysate was examined for hFVIII expression level by ELISA on day 27 post infusion. **c** The phenotypic correction of G-F8-LV treated HemA inhibitor mice (*n* = 7) was examined by tail clip assays on day 160 post infusion. The average blood loss of untreated HemA (*n* = 10) mice was set as 100 %. Wild-type C57BL/6 mice (*n* = 8) were used as positive controls. * *P* < 0.05, ** *P* < 0.005. This figure is reproduced from Ref [10]

Following IO delivery, up to 3 % of platelets expressed FVIII driven by platelet-specific promoters [10] (Fig. 3a). The average hFVIII antigen level evaluated by ELISA was 1 mU per 1 × 10^8^ platelets, a level comparable to previously described transgenic animals expressing platelet FVIII by chromogenic assay [35]. Our results clearly showed that Gp1bα specifically drives FVIII expression in platelets following IO infusion and that FVIII stored in platelets corrects the HemA phenotype. It should be noted that platelet delivered FVIII may produce different kinetics of clot formation temporally and spatially from plasma FVIII as see in the laser injury mouse model [38]. However, enhancement of clot formation with platelet-targeted gene therapy has been demonstrated previously using a range of injury models including tail bleeding assay, survival assay, digital cuticular bleeding assay, and ferric chloride-induced arterial injury model [31, 33–35]. Our recent results [10] clearly showed that even low levels of FVIII released from platelets could partially correct the HemA phenotype. Incorporation of variant FVIII cDNAs with higher expression levels including codon optimized FVIII [24, 25, 39] may further increases the therapeutic effect of IO delivery targeting platelets. Additionally, although platelet-stored FVIII is proven to restore hemostasis, the mouse models have their limitations. Human clinical trials will be the ultimate demonstration that a reduction in incidence of spontaneous bleeds (such as joint bleeds) can be achieved.

### FVIII ectopically expressed and stored in platelets corrects HemA mice with pre-existing high-titer inhibitor antibodies

Much effort has been devoted to investigate effective means to overcome the complication of inhibitory antibody formation to FVIII, including the development of immune modulation protocols (see recent Review [40]) or generation of tissue-specific vectors (including promoter and envelope) or delivery of vectors via specific route [41, 42] to induce tolerance to FVIII. Shi and colleagues [33, 43] reported that pre-conditioning and ex vivo gene transfer of HSCs expressing platelet FVIII improved hemostasis in HemA mice with pre-existing inhibitory antibodies. Our investigation [10] demonstrates that a single IO delivery of G-F8-LV generated significant functional FVIII activity (Fig. 3b). Most importantly, persistent, partial therapeutic effect was obtained despite of the presence of pre-existing high-titer anti-FVIII antibodies (Fig. 3c). These results indicate that FVIII ectopically expressed and stored in α-granules of the platelets and released at the injury sites represents a promising strategy to HemA patients with high-titer inhibitors, including individuals who were previously excluded from gene therapy clinical trials.

### Use pharmacological approaches to enhance in situ transduction efficiency of HSCs following IO delivery of LVs into the BM

Furthermore, following IO delivery of LVs in immune-competent mice, we found lymphocyte infiltrates in BM and gradual decreases of transduced cells, which are similar to the innate and adaptive responses observed from LV transduction in hepatocytes [44–46]. By administering a short course of combination drug treatment with Dexamethasone (Dex) and AntiCD8α antibody, we were able to achieve better initial transduction and reduce the potential of generating CTLs and other adaptive immune responses [47]. These agents in combination synergistically augmented LV transduction.

## Conclusions

Although long-lasting factor concentrates have improved efficacy of protein replacement therapy, and FVIII-mimetic bispecific antibody is promising as an alternative treatment especially for patients with inhibitory antibodies, these therapies require frequent, at least weekly dosing of costly reagents. With the recent successes in clinical trials of AAV-mediated gene transfer, it is anticipated that gene therapy will be the next generation medicine for hemophilia treatment. The potential obstacles of AAV-mediated gene therapy include unpredictable events of transaminase elevation, potential requirement of repeated treatment that cannot be performed due to pre-existing anti-AAV immune responses, and the prohibitively high cost of high-titer vectors needed for hemA treatment. The recently developed novel strategy of IO delivery of F8-LVs [10] can efficiently transduce bone marrow cells and express FVIII in HSCs without the requirement of using potentially toxic, myelosuppressive pre-conditioning methods and the risk of inducing thrombocytopenia. Although ubiquitous expression of FVIII and secretion into the circulation induced high-titer inhibitory antibody production and eliminated functional FVIII activity, a single IO infusion of G-F8-LVs driven by a megakaryocyte-specific promoter produced long-term stable expression of hFVIII in platelets and corrected hemophilia phenotype for long term. Most significantly, this strategy is proven successful in HemA mice with pre-existing inhibitory antibodies. Use of combination of pharmacological agents further enhanced the persistence of transduced cells and transgene expression. IO delivery is already a clinically proven method for drug delivery. IO LV delivery can be easily developed for human clinical trials for hemophilia patients with and without pre-existing inhibitory antibodies. This new simple novel protocol has the potential for a single treatment to achieve life-long therapeutic effect for hemophilia.

## Abbreviations

FVIII (F8 in constructs): Factor VIII
HemA: Hemophilia A
HSC: Hematopoietic stem cells
BM: Bone marrow
IO: Intraosseous
LV: Lentiviral vector
EF1α: Human elongation factor 1 alpha
Gp1bα: Glycoprotein 1b alpha
WAS: Wiskott-Aldrich syndrome
MLD: Metachromatic leukodystrophy
